# Exploring implementation and sustainability of a community paramedicine model to reduce hospitalizations: a pragmatic randomized trial

**DOI:** 10.1186/s12913-026-14532-z

**Published:** 2026-04-17

**Authors:** Jennifer L. Ridgeway, Michelle A. Lampman, Olivia A. Haugo, Amy Glasgow, Jamie L. Smith, Terri L. Menser, Wendy J. S. Sundt, Tami S. Krpata, Michael B. Juntunen, Chad P. Liedl, Joseph G. Hentz, Jessica J. McCoy, Allison Ducharme-Smith, Rozalina G. McCoy

**Affiliations:** 1 Robert D. and Patricia E. Kern Center for the Science of Health Care Delivery, Mayo Clinic, Rochester, MN USA; 2 Research Services – Clinical Trials Office, Mayo Clinic, Rochester, MN USA; 3 Mayo Clinic Ambulance, 200 First Street, Rochester, SW, MN 55905 USA; 4 Quantitative Health Sciences, Mayo Clinic, Phoenix, AZ USA; 5 Division of Community Internal Medicine, Geriatrics, and Palliative Care, Department of Medicine, Rochester, MN USA; 6 Division of Endocrinology, Diabetes, and Nutrition, Department of Medicine, University of Maryland School of Medicine, Baltimore, MD USA; 7 University of Maryland Institute for Health Computing, North Bethesda, MD USA; 8 Center for the Science of Health Care Delivery, Mayo Clinic, 200 First Street, SW, Rochester, MN 55905 USA

**Keywords:** Paramedics (D017051), Delivery of health care (D003695), Patient-centered care (D018802), Implementation science (D000077626), Pragmatic clinical trials as topic (D064792)

## Abstract

**Background:**

Community paramedicine models of care may reduce health system burdens and improve access to care, especially in rural areas. Assessment of intervention implementation from the perspective of implementers and users is critical to refine further implementation, support scale-up, and realize sustainability.

**Methods:**

The parent study was a point-of-care pragmatic randomized trial of a community paramedic (CP) program for patients with intermediate acuity needs (the Care Anywhere with Community Paramedics [CACP] program) versus usual care with the goal of reducing 30-day acute care use. The hybrid effectiveness-implementation design included assessment to explore barriers and facilitators to CACP. Patients in the intervention arm were recruited to complete a survey after the last CP visit, and a sample was invited to an individual interview. All CPs and a sample of clinicians and administrators, purposefully sampled to represent a range of roles and settings with experience using the program, were invited to complete an interview and survey at the end of enrollment, which included questions about program processes, satisfaction with care, and perceptions of CACP sustainability. Surveys were analyzed descriptively, and qualitative data were analyzed using methods of thematic analysis guided by implementation constructs.

**Results:**

Between January 2022 and February 2023, 240 patients were enrolled in the trial; 91 of 119 patients in the intervention arm completed a survey after their last CP visit and 22 were interviewed. Surveys were completed by 63 staff, and interviews were completed by 20 staff. Clinicians reported being very satisfied with communication with CPs (*n* = 34, 82.9%) and implementation of care plans (*n* = 33, 78.6%). Patients also reported high satisfaction, including with CP ability to answer their questions or connect with the right health care provider (*n* = 35, 59% Strongly Agree). Staff impressions of program sustainability varied, though, and included CP concerns about the complexity of some referred patients and insufficient time to prepare for visits.

**Conclusions:**

The program was successfully implemented in busy clinical settings with high levels of overall satisfaction. While feasible and acceptable, additional staffing, refinement of eligible patient populations, and continued improvements to referral and communication systems may be needed to support program sustainability.

**Trial registration:**

ClinicalTrials.govNCT05232799. Registered on 10 February 2022.

**Supplementary Information:**

The online version contains supplementary material available at 10.1186/s12913-026-14532-z.

## Background

Health systems deploy a number of home- and community-based healthcare delivery models to meet patient needs outside brick-and-mortar facilities and increase access to care. These include episodic care models such as transitional care programs and longitudinal models like home-based primary care [1]. Community paramedics (CPs) are emergency medical professionals, typically available through ambulance services, with specialized training in preventive and chronic disease management [2, 3]. Community paramedicine models of care may reduce health system burdens and improve access to care [4, 5] by providing preventive and intermediate levels of care, e.g., infusions and medication management, advanced self-management education, and wound care in community and home settings, either for an episodic need or over multiple visits. They may play an especially important role in rural communities, where access to care is less than in urban areas [6]. Rural clinic, hospital, home health, and skilled nursing facility closures may compound disparities in access to acute and post-acute care [7–10].

The most common goals of CP models include reducing avoidable emergency department (ED) use and hospital readmission, but there is significant heterogeneity of outcome measures and program components [11]. Furthermore, more high-quality randomized trials are needed to evaluate CP program effectiveness [3, 11]. A systematic review published in 2024 found only four randomized trials of mobile integrated health and community paramedicine programs; among those, three evaluated the same primary care services/health education intervention and one a diversion intervention that randomized patients to 12 months of CP support in the home setting or usual care [10].

Research on how community paramedicine models can be feasibly implemented in real-world settings are also needed to ensure effective models are acceptable, sustainable, and equitable [12]. Implementation science frameworks can guide attention to potential implementation determinants relevant to healthcare systems, clinicians, and patients. Even in situations where evaluation of a program’s effectiveness requires a randomized trial design due to lack of robust evidence, embedding implementation assessment during the trial can yield important insights into the factors related to successful implementation, including issues of feasibility and acceptability that underpin the sustainability of new care models [13]. They may be especially important for exploring barriers and facilitators to the implementation of care delivery models that span home and clinical practice settings, where early identification of implementation issues can guide further implementation and sustainability.

This manuscript reports on implementation and sustainability outcomes of a pragmatic trial of a community paramedicine model of care for adults with intermediate care needs seeking to reduce ED and hospital utilization [14]. Although the parent trial found no difference in 30-day acute care use between usual care and CACP, participants reported high satisfaction and a strong preference for receiving future care from community paramedics in the home [15]. This apparent discordance between utilization outcomes and patient experience underscores the need to understand how CACP was implemented in routine practice and what contextual factors may have shaped care delivery and outcomes, particularly given the substantial heterogeneity of the study population and care needs.

## Methods

### Intervention

The Care Anywhere with Community Paramedics (CACP) program is a community paramedicine model of care offered by Mayo Clinic Ambulance. CPs in the program previously delivered care to patients with heart failure and diabetes in the home setting [16, 17], as well as to individuals facing challenges to healthcare access in the community setting [18, 19]. This study established services for patients with intermediate acuity care needs, including development of clinical referral procedures. The CACP scope of service included, but was not limited to, administration of intravenous fluids and diuretics, medication and self-management education, wound care, laboratory specimen collection, and home safety evaluation. Visits were generally limited to two or fewer per day based on program capacity [14]. Referring clinicians specified the services, treatments, and tests needed by the patient in a clinical note within the electronic health record (EHR); additional clarification was obtained through in-basket messages or direct communication, if needed. CPs documented all care within a common EHR, routing their documentation and any questions, concerns, or requests to the patient’s treating clinician (usually the primary care clinician or relevant specialist overseeing care in the outpatient setting). CPs directed questions first to the patient’s treating clinician and then to the CP medical director. Patients were not charged for CP services, and there was no insurance requirement for enrollment.

### Participants and study design

This was a point of care (POC) pragmatic trial, which is pragmatic trial embedded in clinical practice, conducted at Mayo Clinic (Rochester, MN, USA) and the northwest Wisconsin service area of the Mayo Clinic Health System (Barron, WI, USA) [14]. In this study, POC patient recruitment to CACP was with clinical teams in the pre-hospital setting or in the acute care setting (i.e., ED or hospital) as part of routine clinical workflows. Eligible patients were adults with intermediate care needs that could be managed in the home setting with CP support, and they were randomized 1:1 at the POC to usual care or CACP. Using principles of pragmatic trial design, patient eligibility exclusions were minimized and included recommendation from the treating care team that the patient required hospital or ED care that could not be safely delivered by CPs or was otherwise unsafe for CP care at home, inability to provide patient informed consent, home conditions that were unsafe for CPs, prior trial enrollment, and ineligibility based on the service area or residency at a skilled nursing facility.

The trial had a hybrid type 1 effectiveness-implementation study design. Hybrid study designs are used to evaluate both effectiveness and implementation outcomes concurrently. They vary in their relative emphasis on effectiveness versus implementation depending on the existing evidence base for the intervention and its readiness to be evaluated in real-world settings [13, 20]. Hybrid type 1 effectiveness-implementation studies are utilized when the emphasis is on developing evidence of whether the intervention works while simultaneously generating robust information on how to make it work in practice.

There were both effectiveness and implementation outcomes for this trial, which are reported separately. The primary effectiveness outcome of this trial was healthcare utilization, defined as the number of days alive outside the ED or hospital within 30 days of the day following randomization [15]. The implementation outcomes were guided by the Reach, Effectiveness, Adoption, Implementation, Maintenance (RE-AIM) framework [21–24]. This manuscript reports findings related to how the intervention was implemented in routine practice, along with perceptions of its future sustainability (i.e., maintenance). This study was preregistered on ClinicalTrials.gov (NCT05232799) and approved by the Mayo Clinic Institutional Review Board (IRB# 21-010816).

### Data collection

Implementation and maintenance outcomes were assessed using a combination of data from the EHR, an administrative database, surveys, and interviews. Patient experience in this report focuses on that of participants in the intervention group. While the trial included patients randomized to CACP and patients randomized to usual care, implementation evaluation was focused on how CACP was implemented by those who were involved in it; thus, patient participants were limited to those in the CACP intervention group. CPs, clinicians, and administrators were selected based on CACP experience, but they also provide care or are involved in non-CACP care delivery as well.

#### EHR and CP operational database

Baseline patient demographics and orders were abstracted from the EHR. The number of visits and time to visit were also recorded in a CP operational database.

#### Surveys

All patients in the intervention arm were recruited to complete a paper survey at the last CP visit. Surveys were distributed by CPs at the last CP visit, and patients were given the option of returning it in a sealed envelope or completing the survey by telephone with a member of the study team. At the end of the enrollment period, all CPs and all clinicians (physicians, advanced practice professionals, nurse case managers, and social workers) who placed an order for CP care through the CACP program, or provided primary care, or were the assigned inpatient clinician to at least two patients who were enrolled in the program, were invited by email to complete an electronic survey. The study team also identified a purposeful sample of administrators in both geographic regions who were knowledgeable about the CACP program and invited them by email to complete the electronic survey. Patient surveys were conducted from 2/2022 to 4/2023. The CP, clinician, and administrator surveys were conducted from 1/2023 to 3/2023. Two reminder emails were distributed at weekly intervals following the initial contact.

Patient surveys included 17 questions about CP care, communication, and care coordination, as well as questions about satisfaction with care (Supplementary File 1). The CP survey included 9 items focusing on impressions of the CACP program and its processes, along with 10 items dedicated to specific types of clinical and community support. The clinician survey included 11 items focusing on impressions of the CACP program and its processes, as well as 5 items about satisfaction and patient benefit. The administrator survey consisted of 10 items that explored impressions of the CACP program and the perceived benefits for patients. The CP and administrator surveys also included 35 items spanning seven domains of the Clinical Sustainability Assessment Tool (CSAT), a validated measure of perceptions of intervention sustainability: engaged staff and leadership, engaged stakeholders, organizational readiness, workflow integration, implementation and training, monitoring and evaluation, and outcomes and effectiveness [25]. The CSAT rates each item on a 7-point scale from 1= “to little or no extent” to 7= “to a very great extent.” The clinician survey included a shorter set of 15 items in three domains (organizational readiness, workflow integration, and outcomes and effectiveness) based on a requirement to minimize survey burden. All surveys included open-ended questions related to areas for improvement and examples of successes and challenges (Supplementary Files 2-4). There was no participant remuneration for surveys.

#### Interviews

All CPs and eligible patients, as well as a sample of clinicians and administrators, were also invited to complete an individual interview about their experience (Supplementary Files 5-7). Patient interviews were conducted from 7/2022 to 3/2023. The CP, clinician, and administrator interviews were conducted from 1/2023 to 3/2023. Clinician eligibility was based on the same criteria described above for surveys; among eligible clinicians, a purposeful sampling approach was used to identify clinicians representing a balance of roles (e.g., physician, advanced practice professionals) and settings (e.g., primary care, hospital). Clinicians were iteratively recruited in waves, prioritized by numbers of orders, until recruitment targets were achieved and data were sufficient to answer the study questions. Administrators were identified using the same criteria as for surveys. Both clinicians and administrators were invited by email to participate. Patients were eligible if they had completed at least one visit with a CP, had completed their last visit in the CACP program, and were not seriously ill or hospitalized at the time of interview recruitment. All patients meeting these criteria were invited by telephone to participate. The interview guide topics reflected those in the surveys, including satisfaction, care quality and coordination, program sustainability, recommendations for improvement, and CP perceptions of support. Interviews were conducted by phone or using online video conferencing software and were audio recorded and transcribed verbatim for analysis. Interviews were conducted by a member of the study team who has advanced training in qualitative research methods and who was independent of the clinical team. Interview participants were assured of confidentiality as part of the consent process, and individual interview data was not shared with the principal investigator. There was no participant remuneration for interviews.

### Data analysis

EHR data were summarized to describe patient characteristics and CP visits. Surveys were analyzed by role and reported using frequencies. The CSAT was scored by calculating means and standard deviations for each item and domain, where higher scores indicate higher perceived sustainability. Analyses were completed using R software (Version 4.3.2).

Interviews and open-ended survey comments were analyzed using methods of qualitative directed content analysis [26] guided by implementation domains. Two members of the study team reviewed each transcript and coded text using a codebook developed with a priori and emerging topics, including feasibility, acceptability (CP training and skills, continuity of care, communication), satisfaction with care, implementation resources, recommendations for improvement, and perceived sustainability. Coded text was organized using NVivo software (Lumivero, Version 14) to facilitate queries by role and comparison with survey findings.

## Results

Between January 2022 and February 2023, 240 patients were enrolled in the CACP program. Surveys were completed by 63 staff (58% response), and interviews were completed by 20 (mean duration 30 min [SD, 19]), as shown in Table 1. Among patients in the intervention arm (*n* = 119), 91 completed a survey after their last CP visit (77% response; 67 in a sealed envelope and 24 by telephone), and 22 completed an interview (mean duration 21 min, [SD, 12]). Patient survey respondents were mean 67.4 years old (SD, 15.5), 54% female, and 37% rural, and they had a mean 9.4 (SD, 8.2) CP visits while enrolled in the program. Patient interview participants were mean 67 years (SD, 15.8), 59% female, and had a mean 12 CP visits (SD, 8.9).

**Table 1:** Survey and interview participants by role

Role	Survey (*n* =154 )	Interview (*n* = 42)
Administrator	6 (3.9%)	1 (2.4%)
Physician	14 (9.1%)	4 (9.5%)
Nurse practitioner or physician assistant	10 (6.5%)	4 (9.5%)
Case manager or social worker	25 (16.2%)	2 (4.8%)
Nurse manager	1 (0.6%)	3 (7.1%)
Community paramedic	7 (4.5%)	6 (14.3%)
Intervention group patient*	91 (59.1%)	22 (52.4%)

*Patients in the intervention group were recruited to surveys and interviews after the date of service delivery completion

### Barriers and facilitators to implementation

Between January 2022 and February 2023, CPs conducted at least one home care visit with 115 of 119 intervention patients, averaging 8.9 visits (SD, 8.2) per patient, with the first visit a mean 3.5 days (SD, 5.3) from randomization [15]. They executed care plans ordered by treating clinicians, including orders for patient education, wound care, lab collection, and medication reconciliation and education.

#### Perspectives of CPs

Most CPs (*n* = 5/6, 83.3%) agreed or strongly agreed that the process of caring for patients went smoothly. However, only half agreed that the information needed to care for CACP patients was adequately documented in the EHR, and only one-third agreed that orders were adequately documented by the referring clinician or team in the EHR. There was some variability in CP impressions of support by specific members of the care team. Five of the six CPs rated support from patients’ specialty clinicians, on-call clinicians, and social workers moderately high or very high, and four rated support from patients’ primary care providers as moderately high or very high, but four of the six CPs rated support from referring clinicians as somewhat low or very low.

A major theme from interviews and open-ended survey comments was patient complexity. In particular, CPs noted the challenges stemming from the fact that patients referred to the program had a range of healthcare needs, some of which were complex and not understood or known at the time of referral. They also noted that some care was outside their scope of practice and would be more appropriate for a clinician with advanced training.

*The [order] description was*,* sometimes*,* rather brief. They were looking for twice-weekly wound care or daily wound care*,* or they were looking for just some heart failure education…But behind one problem was three problems*,* always and forever. …And I think those problems were not very well-anticipated until we got to the bedside and saw*,* ‘Oh*,* this is what we’re actually dealing with.’…But we kind of have to roll with the punches because*,* at that point in the moment*,* we had already accepted the patient*,* so we were stuck*,* right? CP Interview 5*.

#### Perspectives of clinicians

Clinicians (referring providers, social workers, primary care and specialist providers) reported high satisfaction with various aspects of program implementation, including communication with CPs and CP implementation of care plans, as shown in Table 2.

**Table 2:** Clinician impressions of CP care and program implementation

Satisfaction with…	Very Dissatisfied	Somewhat Dissatisfied	Neither Satisfied nor Dissatisfied	Somewhat Satisfied	Very Satisfied
Communication with CPs	--	--	1 (2.4%)	6 (14.6%)	34 (82.9%)
CPs knowledge and understanding of patient care plans	--	--	1 (2.4%)	5 (11.9%)	36 (85.7%)
Effectiveness of CACP program in meeting patient goals	--	--	1 (2.4%)	8 (19%)	33 (78.6%)
Implementation of patient care plans	--	--	3 (7.1%)	6 (14.3%)	33 (78.6%)

In interviews, clinicians noted that the program addressed persistent gaps in care access for patients in need of intermediate acuity care, especially in rural communities. They reported that the ability of CPs to observe patients in the home setting and share information with them helped them deliver appropriate care without needing to see patients in clinic. Some clinicians noted that this aspect of the program overlapped with other initiatives (e.g., remote patient monitoring). For those patients, the CACP program was filling other gaps created by staffing shortages in other areas, e.g., home health services.

*It’s definitely added an extra layer of support. I feel like they do a really good job of communicating with us. Every time they see a patient in their home*,* I’ll get a note right away from whoever saw them to say*,* ‘Here’s what I did. Here’s the care plan. Are you okay with that? Do you have any other thoughts*,* suggestions*,* recommendations?’ And then*,* we sort of communicate and coordinate behind the scenes. So*,* I feel like this has just been an extra layer of support for us. It’s also added some security*,* knowing that*,* ‘Okay*,* if here’s an issue*,* and I can’t do that*,* I can’t get to the person here*,* there’s an opportunity for the paramedics to go out to them.’ –Clinician Interview 3*.

Clinicians described successful methods of communication and coordination, although some did note potential for increased workload as they manage in-basket or other messages from CPs delivering care in the home, as well as issues with CP staffing capacity. They also reported some difficulty integrating the program into some workflows, e.g., generating lab orders for the CP to complete in the home setting.

*And so*,* I get a lot of messages from the paramedics just via the EHR that I’m able to answer throughout the day with concerns they have. And then*,* they also send me their full notes as well with their assessment…I guess the one other thing that could be a problem is the extra messages*,* I mean*,* because it did*,* at times*,* on certain days when there was some things going on with those patients*,* we did get more messages*,* which already increases the burden that we have with the inbox. –Clinician Interview 10*.

#### Perspectives of patients

As shown in Table 3, most patients rated CPs highly on a range of CP behaviors and aspects of care, including care coordination. In interviews, patients reported that CPs connected them with needed services, sometimes quicker than going through traditional avenues to access healthcare in the clinic setting.

**Table 3:** Patient impressions of community paramedics in the CACP program

Involvement	Strongly Disagree	Disagree	Agree	Strongly Agree	Not Applicable
CPs involved me in decisions about my care as much as I wanted (*n* = 91)	5 (5%)	1 (1%)	19 (21%)	64 (70%)	2 (2%)
CPs worked with me to make a plan for managing my health that I could carry out in my daily life (*n* = 90)	7 (8%)	3 (3%)	19 (21%)	55 (61%)	6 (7%)
CPs encouraged me to ask questions about my care (*n* = 90)	5 (6%)	1 (1%)	22 (24%)	60 (67%)	2 (2%)
CPs were able to answer my questions during their visit or help me connect with the right health care provider to answer those questions (*n* = 91)	5 (5%)	1 (1%)	25 (27%)	57 (63%)	3 (3%)
CPs gave me clear instructions on who to call if I had certain symptoms or side effects (*n* = 89)	5 (6%)	2 (2%)	22 (25%)	53 (60%)	7 (8%)
CPs seemed to work well together as a team (*n* = 89)	5 (6%)	0 (0%)	23 (26%)	56 (63%)	5 (6%)
CPs knew important information about my medical history and the care I needed to receive while enrolled in this program (*n* = 90)	6 (7%)	2 (2%)	25 (28%)	53 (59%)	4 (4%)

Abbreviations: community paramedic = CP; Care Anywhere with Community Paramedic=CACP

*And there were times when I felt like I wasn’t doing so well. I wasn’t getting that water weight off. My chest pressures were still really bad. She contacted the doctor and was able to make those adjustments to my medications so that I did start feeling better. – Patient Interview 152*.

*He was able to come and*,* again*,* give me the IV. And then*,* he came – he said*,* ‘I’ll check again.’ He went on his computer*,* and he got a Mayo Clinic doctor to help make the decision of what to do. They made that decision together for the IV. And then*,* later in the afternoon*,* I think they made the decision again together for the emergency room*,* but I can’t remember. All I remember was that he did include the Mayo Clinic doctor*,* and I saw him on the video on the telehealth kind of thing. And that was nice*,* too*,* to have that extra set of eyes. – Patient Interview 167*.

Some patients noted that scheduling availability was occasionally limited because of staffing, and there were times when CPs were behind schedule. Likewise, there were patients who reported, on surveys, being unsatisfied with care coordination or continuity of care in the program, and interviews provided examples of challenges or misunderstanding related to care expectations. Likewise, patients with complex needs reported wanting reassurance that CPs were aware of their health history and care needs.

*So*,* they kind of dropped the ball on me. They were supposed to come together each week and do whatever it is they were supposed to do. And then*,* they started alternating; one would come one week*,* and the other one would come the next week. As far as getting them to both come*,* it was about impossible. – Patient Interview 213*.

*I do have a complex history*,* and it just – it seems like no one even looks back what I’ve had or what could happen. So*,* that’s what’s really disturbing and really kind of upsets me. –Patient Interview 182*.

### Barriers and facilitators to future sustainability/maintenance

The CSAT assesses perspectives in key domains of sustainability of clinical practices. As shown in Table 4, the mean CSAT score for participants in this study was 5.49 overall (7-point scale: 1 = To little or no extent to 7 = To a very great extent). The highest domain score was in the *Outcomes and Effectiveness* domain (5.9), which includes perceptions of clinically meaningful outcomes and practice advantage. It was lowest in the *Organizational Readiness* domain (5.1), which includes available organizational staffing and resources, although the differences in mean scores were not significant, and all domain scores were above 5.00.

**Table 4:** Clinical sustainability assessment tool scores by participant role

Domain and Sub-domains	Community paramedics (*n* = 7)	Clinicians* (*n* = 50)	Administrators (*n* = 6)	All respondents (*n* = 63)
	Mean (SD)	Mean (SD)	Mean (SD)	Mean (SD)
**Organizational Readiness**	**4.5 (1.2)**	**5.2 (1.0)**	**5.0 (1.0)**	**5.1 (1.0)**
Organizational systems are in place to support the various needs of the CACP program	3.3 (1.3)	5.1 (1.3)	5.2 (1.9)	4.9 (1.4)
The CACP program fits in well with the culture of the team	6.0 (0.8)	6.4 (0.8)	6.5 (0.6)	6.4 (0.8)
The CACP program has feasible and sufficient resources (e.g., time, space, funding) to achieve its goals	4.0 (1.6)	4.7 (1.5)	3.7 (1.2)	4.5 (1.5)
The CACP program has adequate staff to achieve its goals	4.5 (1.0)	4.6 (1.3)	3.7 (1.2)	4.5 (1.3)
The CACP program is well integrated into the operations of the organization	4.5 (2.7)	5.1 (1.5)	5.8 (1.0)	5.2 (1.5)
**Workflow Integration**	**5.3 (0.7)**	**5.7 (0.9)**	**5.5 (1.0)**	**5.7 (0.9)**
The CACP program is built into the clinical workflow	4.5 (1.0)	4.9 (1.5)	5.0 (1.4)	4.9 (1.2)
The CACP program is easy for clinicians to use	4.0 (0.8)	5.7 (1.3)	6.0 (1.2)	5.6 (1.4)
The CACP program integrates well with established clinical practices	5.3 (1.7)	5.9 (1.0)	5.7 (1.4)	5.8 (1.1)
The CACP program aligns well with other clinical systems (e.g., electronic health record)	5.3 (1.7)	6.1 (1.0)	5.7 (1.9)	6.0 (1.2)
The CACP program is designed to be used consistently	4.8 (1.7)	5.7 (1.3)	5.5 (0.8)	5.6 (1.3)
**Outcomes & Effectiveness**	**4.4 (1.1)**	**6.2 (0.0)**	**5.6 (1.3)**	**5.9 (1.1)**
The practice has evidence of beneficial outcomes	5.0 (0.0)	6.2 (1.1)	5.7 (1.8)	6.0 (1.2)
The practice is associated with improvement in patient outcomes that are clinically meaningful	4.8 (0.5)	6.3 (0.9)	6.5 (0.8)	6.2 (1.0)
The practice is clearly linked to positive health or clinical outcomes	4.0 (1.2)	6.1 (0.9)	6.3 (1.2)	6.0 (1.1)
The practice is cost-effective	4.0 (1.8)	6.0 (1.1)	5.0 (1.6)	5. 7 (1.4)
The practice has clear advantages over alternatives	4.0 (2.3)	6.4 (0.7)	4.6 (1.1)	6.0 (1.3)
**Overall Mean by Group**	**4.3**	**5.6**	**5.4**	**5.5**

There was some variability by participant role. CP scores were lowest overall and on items related to perspectives of organizational readiness, especially perspectives on the extent to which organizational systems were in place to support the various needs of the CACP program (3.3). Perspectives on the availability of organizational staffing and resources were low across groups and lowest for administrators. Clinician and CP perspectives on the extent to which the CACP program was built into the clinical workflow were also lower. Perspectives of the extent to which the CACP program fit in well with the culture of the team was high across all participant groups.

Interviews further highlighted opportunities and challenges related to future sustainability or scale-up of the program. Principally, while the program was considered to be effective at addressing a critical care gap, CPs, in particular, perceived the care needs of highly complex patients to be difficult to sustain given current levels of staffing and training. Clinicians also reported the need for leadership support and financial support for sustainability.

CPs noted areas where additional specialized training would help prepare them to deliver care, but CP and clinician interviews also suggested that sustainability hinges on improved staffing for the program, as well as better availability of other clinical and community health care resources. Both CPs and clinicians reported additional wound care providers are needed, and CPs indicated the need for greater access to social workers to get patients connected with community services and to address health-related social needs.

Recommendations for program improvement to support sustainability, identified in interviews, are shown in Table 5, organized by CSAT domains. They include recommendations related to patients, CPs, clinicians, and organizational or health system factors. The greatest number of recommendations were in the domain of organizational readiness.

**Table 5:** Recommendations for improvements to bolster CACP sustainability

Domain	Recommendation	Exemplary Quotes
Organizational readiness (i.e., sufficient staff and other resources), especially for complex patients	Hire additional staff and provide time for CP appointment preparation	“Because I’m sure they have staffing problems like everything else. And if there’s times where their staffing can’t – is the limitation to accepting a patient, that just impacts buy-in.” *Clinician Interview 8* “I was drowning here…I had unpredictable scenarios that kept popping up throughout my day. I was 2 ½ hours behind schedule. There’s nobody else. There’s nobody here that can help me when it gets to that point…I had a 4:00 appointment in [city], so I’m not going to make it down there by any means nor have I had time to prep for this patient’s graduation.” *CP Interview 2* “I think [additional hiring] will definitely help with the demand that we have from patients for us and to be able to give even more like individualized attention, even though – I mean we do, but the days end up being very long – longer than I had anticipated, especially when I work operations. Usually, I’m there 14 or 15 hours a day for a 12-hour shift because that’s how it works out in order to see all the patients that I’m seeing, get all of their documentation done, reach out to the folks that I need to as far as additional resources for whoever, whether that’s pharmacy, the county, whatever that might be.” *CP Interview 3*
	Limit patient inclusion criteria in terms of complexity and improve clinician communication/ handoffs to describe the full range of patient needs at referral	“Sometimes I feel like community paramedics can become a little bit of a dumping group for patients that aren’t getting or have been denied by other resources. So, I just think that for their processes and flows and looking into the future of how they’re going to meet patient needs just to kind of develop some inclusion criteria so that they don’t feel like they’re getting these high-complexity patients that have a lot of psychosocial and medical conditions but also and then take up a lot of their time. I think just having a little bit more streamlined inclusion criteria could help that but also not like excluding patients based off of being difficult.” *Clinician Interview 3* “So, I think, going forward, we’re going to have a ton of referrals. I think what we need to do is hone in on what we’re really, really good at; or like we need to establish like some clear parameters for like which patients will fall into our program… CACP was great because we were a catch-all for anything and everything. But we – this, hopefully, will help us identify some things we were unsuccessful for, and then maybe those aren’t the right patients for us. So, that’s what I’m kind of hoping is that we’ll figure out like, ‘Hey, we had a real big impact on these kind of patients, not so much on these; let’s focus on what we’re having better outcomes with or what we’re like actually positively impacting.’” *CP Interview 4*
	Provide additional training for CPs to support specialties	“It probably will be something whereby the community paramedics will probably end up needing almost subspecialty like training for each of the different disease states that they’re managing…If they start doing end-stage liver disease with the hepatology team, maybe they’ll have to know how to do a home paracentesis.” *Clinician Interview 1* “…I think, to really, really have a consistent grasp of some of these complex topics, we need to spend time inpatient with community internal medicine or diabetic educators or nutritionists. You can’t just read it in a book and try to teach patients about it or colostomy bag cares.” *Community Paramedic 6*
	Maintain leadership support and make coverage details clear for patients	“I will say if there’s a challenge that we have had in our regions, in particular, it has been overcoming some of the billing nuances so that we can bill for these.” *Administrator 13* “I love that we’re not billing patients [during the trial]. So, I’m kind of nervous, going forward, how that’s going to change our relationship with patients.…And I think when they’re getting a bill, I think it’s going to be more important for us to provide a much higher standard and polished level of care than we have, at times, given our patient load with just lack of time for case preparation or just how busy our schedule is…” *CP 6* “I would say, the big things are just continued support from our leadership, continued support from our medical director, and additional services that we converse with quite a bit are kind of the big things. Money is always a thing – that the funding is there.” *CP 3*
Workflow integration (i.e., integration into clinician practices and electronic systems and workflows)	Develop systems for efficient care team support and communication	“I would also recommend that you have a really well-streamlined line of communication to your provider’s team so that you can work through problems because a big hiccup, like I said, we had was dealing with providers, nonresponsive providers or having ill-defined care plans. So, I would say be ready to educate yourselves and be ready and be a staunch communicator as that is going to be about 90% of what your treatments will involve will be communication of problems to the right people to solve them, if you can’t solve it yourself.” *CP 5* “I think the more providers know about what we’re doing, the more they can communicate and like reassure the patient their faith in us and our program because you really did notice when we had patients who had worked with providers that worked with us a lot, the transition was smoother because they were really able to prepare their patient and explain why this was of value.” *CP 4* “I think, having a specific point of contact with a phone number off hours and during hours that we can reach with questions, because having [medical director] try to follow up, especially – because I work weekends. I don’t have any regular point of contact that I can reach out to. I can’t message providers in Epic and expect any answer during my Saturday/Sunday community paramedic shifts that I work. I work every Saturday/Sunday as a CP. So, assigning like a case manager, someone, like I said, at least 7:00 a.m. to 7:00 p.m., seven days a week that we can touch base that knows about these cases, and I think that’s kind of coming, going forward with the CP service because we know there’s a need.” *CP 6*
	Coordinate with other health and social service providers to limit duplication	“I think there’s probably a place to partner with our other primary care programs so that there is less duplication and more of a working together or looking at patients differently in the fact that they need help with X, Y, and Z. They could have community paramedics help with this piece and then another program to help with this piece and not so much of a, “This patient doesn’t qualify for this program, so then, the next thing we’re going to go to is community paramedics.” *Clinician 3*
Outcomes and effectiveness (i.e., clear advantages over alternatives and evidence of outcomes)	Need to demonstrate program value to decision makers for support	“I don’t know. I’m not sure. They sound supportive. I mean they haven’t shut it down yet, right? And I think that’s where the research is going to be very helpful, too, is to take a population that we’ve worked with so far, show cost savings, health outcomes, qualitative analysis of what providers think, just like what we’re doing right now, how we’ve been able to benefit that, and to put all that together to show overall value, right? It’s all about value to patients, providers, health care system.” *Clinician 12* “Hands down, this program, to me, is very much needed in our community, and I think in order for it to continue to be successful is we need to assure that the paramedics have all the resources that they need, if they need something.” *Clinician 6*
	Be intentional about scaling up services	“We ought to align ourselves with this program and determine what services could potentially be expanded or scaled further versus which ones we feel can just continue to grow organically, so there’s a little intentionality about what we do. And if we try to do everything for everyone, I think, particularly when we’ve got some resource challenges or whatever, we’re going to suboptimize overall performance.” *Administrator 13*

Abbreviations: community paramedic = CP; Care Anywhere with Community Paramedic=CACP

## Discussion

This study found high levels of satisfaction with the program, including high levels of satisfaction with CP care, communication, and care coordination across all participant groups. Clinician satisfaction, in particular, may have stemmed from the fact that resources for patients with intermediate acuity care needs (e.g., intravenous medications, wound care) are a critical gap in the regions of this study, especially rural communities in the catchment area. Several alternative workforce strategies have been proposed to address rural health workforce shortages, including community health workers (CHWs), telehealth-integrated care coordination, and expanded home health services. Compared to CHWs, who typically focus on health education, social needs navigation, and care coordination for patients with chronic conditions, the CP model is better suited to patients requiring clinical assessment, clinical intervention, medication management, more advanced education, and skilled procedural care (e.g., wound care, intravenous medications) that fall outside the CHW and even home health nursing scope of practice. However, CPs require extensive training and credentialing that is not available in all jurisdictions, meaning training, skills, and experience may be inconsistent and, in some cases, insufficient for this scope of practice.

This study also identified challenges with implementation of CACP in routine clinical practice. A key feature of hybrid type 1 effectiveness-implementation studies is that assessment of implementation in routine care can help explain effectiveness outcomes, including in randomized trials [13, 20, 27]. In this study, findings around broad inclusion criteria, as well as limited clinician awareness of the scope of practice, may help explain why we found no difference between CACP and usual care in 30-day acute care use [15]. Another key feature is that hybrid type 1 effectiveness-implementation studies identify contextual factors related to implementation success [13, 20]. This study identified key recommendations, including factors related to staff satisfaction with implementation. These include the improvements needed for referral, documentation systems, as well as communication and support from care teams. CPs, in particular, reported needing adequate information in the EHR to deliver care, as well as ready support from care teams to manage complex patient needs. As home-based models like this continue to proliferate, robust documentation and communication systems are needed to ensure patient continuity of care and to support healthcare workers at the POC.

Previous work has reported that expanded services and training are factors CPs perceive as related to sustainability [28]. While clinician satisfaction may have been related to expanded services for their patients, there were also challenges related to complexity of patient needs. The pragmatic design of this trial expanded early pilot studies of diabetes and heart failure care to make the model available to a broad range of patients and make program referrals available to clinicians in varied settings [29]. Broad eligibility criteria ensure equitable program access to the patients who need it without restrictions that may disproportionately exclude patients from underserved communities and those facing structural barriers to care. This approach may also benefit smaller clinical settings that may not be able to operationally sustain a narrowly focused clinical program, and it may make the program more appealing to hospital or health system leaders who make funding decisions because of the broad range of care needs that can be met with a single service, which is essential for program launch and sustainability, especially in smaller communities with lower patient volumes. Indeed, narrowly defined programs may face sustainability challenges if the clinical priorities or needs shift toward other goals.

Conversely, narrower eligibility criteria enable a more homogenous and predictable patient population, making it easier to develop workflows and more comprehensively train CPs. Varied and complex patient needs created challenges for CPs, who managed a wide array of patient needs, some of which were not clear in the electronic orders and that only became apparent when the CP arrived in the home [29]. Patient complexity may also require additional time for CPs to prepare for and deliver care, as well as additional CP training and time for physicians and other members of the care team to support CPs. Maintaining balance between (1) eligibility criteria that make care accessible and the program fundable and (2) an appropriate and feasible scope of service will be critical for future program success. Lack of sustainable funding is a barrier to stable staffing, with some agencies reliant on overtime personnel for CP coverage.

Strengths of this work include the use of qualitative and quantitative methods and representation of multiple perspectives. Use of implementation constructs to guide data collection and analysis also grounded interpretation of findings within core constructs known to be relevant to implementation and sustainability, e.g., workflow integration and leadership support. The multidisciplinary study team, which included social scientists and clinicians, also served to reduce interpretive bias. Limitations include potential selection bias among interview participants, wherein clinicians and administrators who were most engaged and most supportive of the program may have been more likely to participate in interviews. Likewise, patients with high program satisfaction may have been more likely to participate in an interview, although interview data included less favorable impressions of some aspects of the program. Similarly, while the parent trial surveyed patients in the intervention and control conditions, staff surveys were limited to individuals who were involved in or impacted by the CACP program. While this sampling approach provides rich data on CACP program implementation, future research with a broader group of professionals may be needed to ascertain the relative benefits of this program compared to alternative programs.

Generalizability of this model may also be limited for independent ambulance agencies or municipal fire departments that face greater challenges to leadership support than Mayo Clinic Ambulance, which is part of the Mayo Clinic network. Integration of the ambulance service in the health system network provides advantages, including shared goals and priorities, consistent resource allocation, and a shared EHR to facilitate collaboration and communication. As community paramedicine models become increasingly widespread, it will be important to examine their implementation and sustainability across the full range of their implementation across the U.S.

## Conclusions

The CACP program was successfully implemented in busy clinical settings with high levels of overall satisfaction from patients, clinicians, and CPs. While feasible and acceptable in this trial, additional staffing, refinement of eligible patient populations, and continued improvements to referral and communication systems may be needed to support program sustainability, despite perceptions that the program fills important care gaps in community settings.

## Supplementary Information

Below is the link to the electronic supplementary material.

Supplementary Material 1

Supplementary Material 2

Supplementary Material 3

Supplementary Material 4

Supplementary Material 5

Supplementary Material 6

Supplementary Material 7

## Data Availability

The datasets used and/or analyzed during the current study are available from the corresponding author on reasonable request.

## Abbreviations

CACP: Care Anywhere with Community Paramedics
CP: Community paramedic
CSAT: Clinical Sustainability Assessment Tool
ED: Emergency department
EHR: Electronic health record
RE-AIM: Reach, Effectiveness, Adoption, Implementation, Maintenance
