# Supplementary material for: Exploring implementation and sustainability of a community paramedicine model to reduce hospitalizations: a pragmatic randomized trial
Source: BMC Health Serv Res. 2026 Apr 17;26:763. doi: 10.1186/s12913-026-14532-z (PMC13217778; doi:10.1186/s12913-026-14532-z)
Supplement: Supplementary file 1 — Supplementary Material 1 [file 12913_2026_14532_MOESM1_ESM.pdf]

MAYO CLINIC

Care Anywhere with Community Paramedics Evaluation Survey

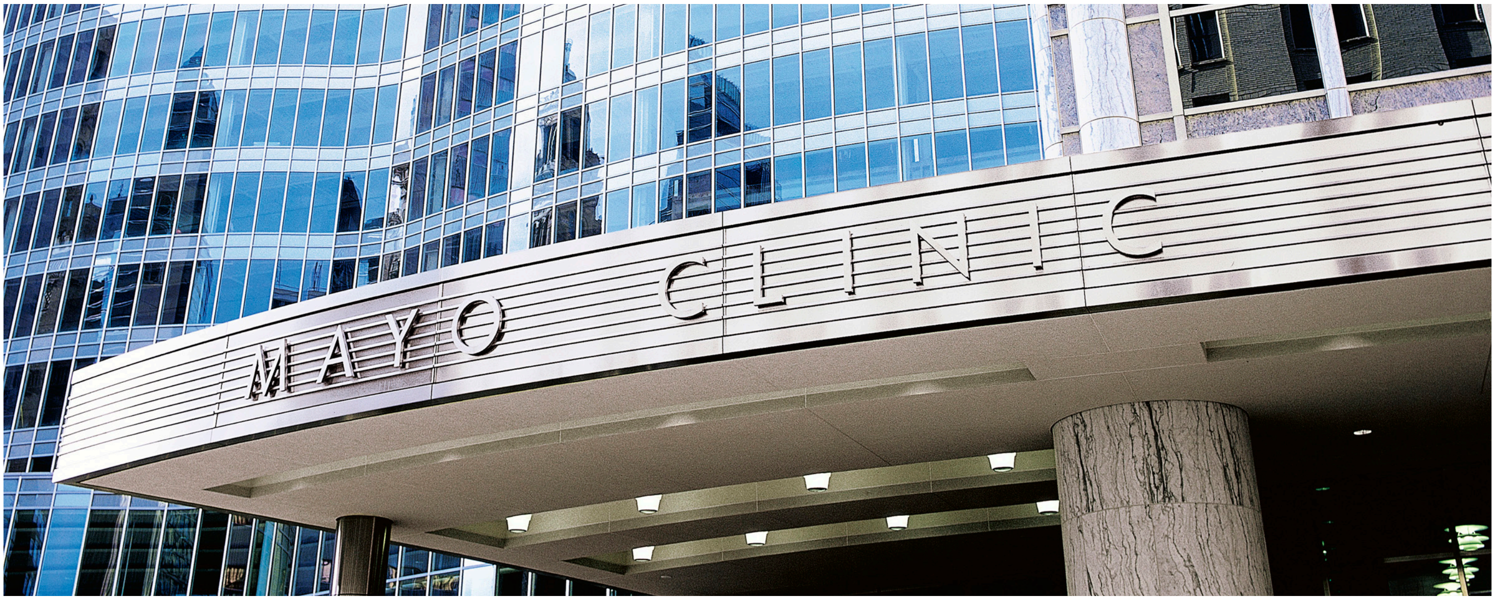

Survey Research Center

Name: ______________________________ _____ ____________________________________
First MI Last

Clinic Number: __ __-__ __ __-__ __ __

INSTRUCTIONS: PLEASE CHECK THE APPROPRIATE BOX OR FILL IN THE BLANK AS INDICATED.

1. **Today's Date:** __ __/__ __/__ __ __ __
   Month Day Year

2. **How confident are you that you are able to successfully manage your medical conditions at home?**

   1 ☐ Not at all confident
   2 ☐ Slightly confident
   3 ☐ Moderately confident
   4 ☐ Very confident
   5 ☐ Extremely confident

ABOUT YOUR EXPERIENCE WITH COMMUNITY PARAMEDIC HOME VISITS

For each item, please mark the box that best describes how you feel or what is true for you.

3. **Involvement in Care**

	Strongly disagree	Disagree	Agree	Strongly agree	Not applicable
**Community paramedics involved me in decisions about my care as much as I wanted.**	1 ☐	2 ☐	3 ☐	4 ☐	5 ☐
**Community paramedics worked with me to make a plan for managing my health that I could carry out in my daily life.**	1 ☐	2 ☐	3 ☐	4 ☐	5 ☐
**Community paramedics worked with my family or caregiver to make a plan for managing my health that I could carry out in my daily life.**	1 ☐	2 ☐	3 ☐	4 ☐	5 ☐
**Community paramedics encouraged me to ask questions about my care**	1 ☐	2 ☐	3 ☐	4 ☐	5 ☐

4. **Communication and Health Information**

	Strongly disagree	Disagree	Agree	Strongly agree	Not applicable
**Community paramedics were able to answer my questions during their visit or help me connect with the right health care provider to answer those questions**	1 ☐	2 ☐	3 ☐	4 ☐	5 ☐
**Community paramedics explained things in a way that was easy for me to understand**	1 ☐	2 ☐	3 ☐	4 ☐	5 ☐
**Community paramedics gave me the skills I needed to recognize certain symptoms and side effects**	1 ☐	2 ☐	3 ☐	4 ☐	5 ☐
**Community paramedics gave me clear instructions on who to call if I had certain symptoms or side effects**	1 ☐	2 ☐	3 ☐	4 ☐	5 ☐
**Community paramedics gave me the skills I needed to take my medications**	1 ☐	2 ☐	3 ☐	4 ☐	5 ☐
**Community paramedics gave me the skills I I needed to use medical equipment or devices such as blood pressure cuff, blood sugar monitor, etc.**	1 ☐	2 ☐	3 ☐	4 ☐	5 ☐

5. **Caring and Concern**

	Strongly disagree	Disagree	Agree	Strongly agree	Not applicable
**Community paramedics treated me with courtesy and respect**	1 ☐	2 ☐	3 ☐	4 ☐	5 ☐
**Community paramedics listened carefully to my questions and concerns**	1 ☐	2 ☐	3 ☐	4 ☐	5 ☐
**Community paramedics were caring and and concerned about me**	1 ☐	2 ☐	3 ☐	4 ☐	5 ☐

6. **Care Coordination**

	Strongly disagree	Disagree	Agree	Strongly agree	Not applicable
**Community paramedics seemed to work well together as a team**	1 ☐	2 ☐	3 ☐	4 ☐	5 ☐
**Community paramedics knew important information about my medical history**	1 ☐	2 ☐	3 ☐	4 ☐	5 ☐
**Community paramedics knew about the care I needed to receive while enrolled in this program**	1 ☐	2 ☐	3 ☐	4 ☐	5 ☐
**Community paramedics seemed to work well together with my other health care providers**	1 ☐	2 ☐	3 ☐	4 ☐	5 ☐

7. **In general, how <u>satisfied</u> are you with the care that you received from the community paramedics?**

1 ☐ Not at all satisfied
2 ☐ Slightly satisfied
3 ☐ Moderately satisfied
4 ☐ Very satisfied
5 ☐ Extremely satisfied

8. **How <u>comfortable</u> were you with receiving care from the community paramedics in your home?**

1 ☐ Not at all comfortable
2 ☐ Slightly comfortable
3 ☐ Moderately comfortable
4 ☐ Very comfortable
5 ☐ Extremely comfortable

9. **How <u>safe</u> did you feel with the care that you received from the community paramedics?**

1 ☐ Not at all safe
2 ☐ Slightly safe
3 ☐ Moderately safe
4 ☐ Very safe
5 ☐ Extremely safe

10. **How likely are you to recommend the community paramedic program to other patients like you for their medical care?**

1 ☐ Not at all likely
2 ☐ Slightly likely
3 ☐ Moderately likely
4 ☐ Very likely
5 ☐ Extremely likely

11. If you are ill again in the future, would you prefer to be treated for your condition in the hospital or at home with community paramedic services?

1 ☐ Strongly prefer to be in the hospital
2 ☐ Somewhat prefer to be in the hospital
3 ☐ Somewhat prefer in-home care with community paramedic visits
4 ☐ Strongly prefer in-home care with community paramedic visits
5 ☐ No preference or not sure

12. How likely are you to recommend Mayo Clinic to others for their medical care?

1 ☐ Not at all likely
2 ☐ Slightly likely
3 ☐ Moderately likely
4 ☐ Very likely
5 ☐ Extremely likely

13. Does having the option for treating your health condition at home with community paramedic support make you more or less likely to choose Mayo Clinic for your medical care needs?

1 ☐ A lot less likely
2 ☐ Slightly less likely
3 ☐ Neither more nor less likely
4 ☐ Somewhat more likely
5 ☐ A lot more likely

14. Please describe anything that you think has gone <u>particularly well</u> in the community paramedic program.

____

____

____

____

____

15. Please describe anything that you think has gone poorly to help us make improvements in the future.

____

____

____

____

____

16. What other resources or support would have been helpful for you to have at this time?

____

____

____

____

____

17. Did someone help you complete this survey?

1 ☐ No   2 ☐ Yes

How did that person help you? (Mark all that apply.)

1 ☐ Read the questions to me
1 ☐ Wrote down the answers I gave
1 ☐ Answered the questions for me
1 ☐ Translated the questions into my language
1 ☐ Helped in some other way, please specify:

____

____

____

____

Thank you for completing this survey!

Please return your completed survey in the envelope provided.

If your envelope is missing, please mail your survey to:

Survey Research Center
Harwick 7
200 First Street SW
Rochester MN 55905

©2021 Mayo Foundation for Medical Education and Research. All rights reserved. MAYO, MAYO CLINIC and the triple-shield Mayo logo are trademarks and service marks of MFMER.

MC4269-000

(THIS PAGE IS FOR SURVEY RESEARCH TRACKING AND FILING PURPOSES ONLY)

CARE ANYWHERE WITH COMMUNITY PARAMEDICS

Evaluation Survey

Investigator: Rozalina McCoy, M.D.

Version as of:
November 17, 2021 cam
December 8, 2021 cam
