# Supplementary material for: Exploring implementation and sustainability of a community paramedicine model to reduce hospitalizations: a pragmatic randomized trial
Source: BMC Health Serv Res. 2026 Apr 17;26:763. doi: 10.1186/s12913-026-14532-z (PMC13217778; doi:10.1186/s12913-026-14532-z)
Supplement: Supplementary file 2 — Supplementary Material 2 [file 12913_2026_14532_MOESM2_ESM.pdf]

INSTRUCTIONS: Please check the appropriate box or fill in the blank as indicated. The following questions refer to the Care Anywhere with Community Paramedics (CACP) program. Patients were those randomized to home care with community paramedic support, after they were referred from the outpatient setting, emergency department, or hospital, as part of a research study. It does not include other community paramedic care services outside of the CACP program. In this survey, "home" refers to any non-clinical location where you cared for patients enrolled in the *CACP* program.

1. What is your primary practice location?
   - ☐ Rochester/Southeast Minnesota
   - ☐ Northwest Wisconsin

Thinking of the patients you cared for in the CACP program, how much do you agree or disagree with the following statements? "H
Instructions: For each item, please mark an "X" in the box that best describes how you feel or what is true for you.

		Strongly agree	Agree	Disagree	Strongly disagree	Not applicable
1.	Patients in the CACP program were well prepared for enrollment in the program	1☐	2☐	3☐	4☐	5☐
2.	Patients referred to the *CACP* program were appropriate for this care delivery model	1☐	2☐	3☐	4☐	5☐
3.	Information needed to care for CACP patients was adequately documented in the electronichealth record	1☐	2☐	3☐	4☐	5☐
4.	Orders needed to care for CACP patients were adequately documented in the electronic health record	1☐	2☐	3☐	4☐	5☐
5.	The process of accepting new patients into the *CACP* program went smoothly	1☐	2☐	3☐	4☐	5☐
6.	The process of caring for patients enrolled in the *CACP* program went smoothly	1☐	2☐	3☐	4☐	5☐
7.	I had the education and training necessary to deliver the care that my CACP patients needed	1☐	2☐	3☐	4☐	5☐
8.	I had the equipment necessary to deliver the care that my CACP patients needed	1☐	2☐	3☐	4☐	5☐
9.	I had the support necessary to deliver the care that my CACP patients needed	1☐	2☐	3☐	4☐	5☐

Role clarity

	Completely disagree	Disagree	Somewhat disagree	Neither agree nor disagree	Somewhat agree	Agree	Completely agree
1. My role as a community paramedic in the CACP program is clearly defined for me.	1	2	3	4	5	6	7
2. I know what my responsibilities are as a community paramedic in the CACP program.	1	2	3	4	5	6	7
3. In my work as a community paramedic in the CACP program, I know exactly what is expected from me.	1	2	3	4	5	6	7

Knowledge and skills

	Completely disagree	Disagree	Somewhat disagree	Neither agree nor disagree	Somewhat agree	Agree	Completely agree
1. I know how to deliver the medical care in the *CACP* program	1	2	3	4	5	6	7
2. I have been trained in the medical care I provide in the *CACP* program	1	2	3	4	5	6	7
3. I have the skills to deliver the medical care I provide in the *CACP* program	1	2	3	4	5	6	7

4. I am practiced to deliver the medical care I provide in the *CACP* program	1	2	3	4	5	6	7

Beliefs about capabilities

	Very difficult						Very easy
1. For me, performing patient assessments as part of the *CACP* program is	1	2	3	4	5	6	7
2. For me, delivering education to patients and caregivers as part of the *CACP* program is	1	2	3	4	5	6	7
3. For me, delivering chronic disease management care as part of the *CACP* program is	1	2	3	4	5	6	7
4. For me, delivering acute illness care as part of the *CACP* program is	1	2	3	4	5	6	7

For the questions below, "home" refers to any non-clinical location where you cared for patients enrolled in the *CACP* program.

Impact on CP: burden and safety concerns

	Completely disagree	Disagree	Somewhat disagree	Neither agree nor disagree	Somewhat agree	Agree	Completely agree
1. I feel a great deal of stress because of my work as a community paramedic in patient homes.	1	2	3	4	5	6	7
2. I am enthusiastic about my work as a community paramedic in patient homes.	1	2	3	4	5	6	7
3. I often worry about my safety when working as a community paramedic in patient homes.	1	2	3	4	5	6	7

How would you rate the level of support you received from these individuals or departments while caring for patients in the *CACP* program?
Instructions: For each item, please mark an "X" in the box that best describes how you feel or what is true for you.

		Very high	Moderately high	Somewhat low	Very low	Not applicable
10.	Referring clinicians	1☐	2☐	3☐	4☐	5☐
11.	On-call clinicians from the referral service (if contacted during or after clinic hours)	1☐	2☐	3☐	4☐	5☐
12.	Patients' primary care providers	1☐	2☐	3☐	4☐	5☐
13.	Specialty clinicians involved in the care of enrolled patients (such as wound care, endocrinology, cardiology, etc.)	1☐	2☐	3☐	4☐	5☐
14.	Mayo Clinic Ambulance Medical Direction (includes Medical Director, Operations Manager, Community Paramedic Coordinator, or another supervisor)	1☐	2☐	3☐	4☐	5☐
15.	TeleEM physicians	1☐	2☐	3☐	4☐	5☐
16.	Pharmacy staff (when picking up medications)	1☐	2☐	3☐	4☐	5☐
17.	Outpatient laboratory staff (when dropping off patient lab samples)	1☐	2☐	3☐	4☐	5☐
18.	Social workers or other clinical social support providers	1☐	2☐	3☐	4☐	5☐
19.	Community organizations or public health agencies	1☐	2☐	3☐	4☐	5☐

In the following questions, please rate the *CACP* program across a range of specific factors that affect program sustainability. Please respond to as many items as possible. If you truly feel you are not able to answer an item, you may select "NA." For each statement, select the number that best indicates the extent to which your practice has or does the following things.

20. **Engaged staff and leadership**: Having supportive frontline staff and management within the program.

	To little or no extent						To a very great extent	Not able to answer
The *CACP*program engages leadership and staff throughout the process	1	2	3	4	5	6	7	NA

Clinical champions of the *CACP* program are recognized and respected	1	2	3	4	5	6	7	NA
The *CACP* program has engaged, ongoing champions	1	2	3	4	5	6	7	NA
The *CACP* program has a leadership team made of multiprofessional partnerships	1	2	3	4	5	6	7	NA
The *CACP* program has team-based collaboration and infrastructure	1	2	3	4	5	6	7	NA

21. **Engaged stakeholders**: Having external support and engagement for the program

	To little or no extent						To a very great extent	Not able to answer
The *CACP* program engages the patient and family members as stakeholders	1	2	3	4	5	6	7	NA
There is respect for all stakeholders involved in the *CACP* program	1	2	3	4	5	6	7	NA
The *CACP* program is valued by a diverse set of stakeholders	1	2	3	4	5	6	7	NA
The *CACP* program engages other medical teams and community partnerships as appropriate	1	2	3	4	5	6	7	NA
The *CACP* program team has the ability to respond to stakeholder feedback about the program	1	2	3	4	5	6	7	NA

22. **Organizational readiness**: Having the internal support and resources needed to effectively manage the program.

	To little or no extent						To a very great extent	Not able to answer
Organizational systems are in place to support the various needs of the *CACP* program	1	2	3	4	5	6	7	NA
The *CACP* program fits in well with the culture of the team	1	2	3	4	5	6	7	NA
The *CACP* program has feasible and sufficient resources (e.g., time, space, funding) to achieve its goals	1	2	3	4	5	6	7	NA
The *CACP* program has adequate staff to achieve its goals	1	2	3	4	5	6	7	NA
The *CACP* program is well integrated into the operations of the organization	1	2	3	4	5	6	7	NA

23. **Workflow integration**: Designing the program to fit into existing practices and technologies.

	To little or no extent						To a very great extent	Not able to answer
The *CACP* program is built into the clinical workflow	1	2	3	4	5	6	7	NA
The *CACP* program is easy for clinicians to use	1	2	3	4	5	6	7	NA
The *CACP* program integrates well with established clinical practices	1	2	3	4	5	6	7	NA
The *CACP* program aligns well with other clinical systems (e.g. electronic health record)	1	2	3	4	5	6	7	NA
The *CACP* program is designed to be used consistently	1	2	3	4	5	6	7	NA

24. **Implementation and training**: Using processes that guide the direction, goals, and strategies of the program.

	To little or no extent						To a very great extent	Not able to answer
The *CACP* program clearly outlines roles and responsibilities for all staff	1	2	3	4	5	6	7	NA
The reason for the *CACP* program is clearly communicated to and understood by all staff	1	2	3	4	5	6	7	NA
Staff receive ongoing coaching, feedback, and training	1	2	3	4	5	6	7	NA
CACP program implementation is guided by feedback from stakeholders	1	2	3	4	5	6	7	NA
The *CACP* program has ongoing education across professions	1	2	3	4	5	6	7	NA

25.

	To little or no extent						To a very great extent	Not able to answer
The *CACP* program has measurable process components, outcomes, and metrics	1	2	3	4	5	6	7	NA
Evaluation and monitoring of the *CACP* program are reviewed on a consistent basis	1	2	3	4	5	6	7	NA
The *CACP* program has clear documentation to guide process and outcome evaluation	1	2	3	4	5	6	7	NA
CACP program monitoring, evaluation, and outcomes data are routinely reported to the clinical care team	1	2	3	4	5	6	7	NA
The *CACP* program process components, outcomes, and metrics are easily assessed and audited	1	2	3	4	5	6	7	NA

26. **Outcomes and effectiveness**: Understanding and measuring program outcomes and impact.

	To little or no extent						To a very great extent	Not able to answer
The *CACP* program has evidence of beneficial outcomes	1	2	3	4	5	6	7	NA
The *CACP* program is associated with improvement in patient outcomes that are clinically meaningful	1	2	3	4	5	6	7	NA
The *CACP* program is clearly linked to positive health or clinical outcomes	1	2	3	4	5	6	7	NA
The *CACP* program is cost-effective	1	2	3	4	5	6	7	NA
The *CACP* program has clear advantages over alternatives	1	2	3	4	5	6	7	NA

27. Could the *CACP* program be improved by expanding types of services community paramedics can provide in the home?
   - ☐ No, current CACP program services are adequate
   - ☐ Yes, the program would be improved if services were expanded
     Please specify what services could be added

     ____

28. Do you have any concerns about the process or timing of patients' discharge from the hospital or emergency department to the CACP program?

- ☐ No
- ☐ Yes (specify):

  ____

29. Please describe anything that you think has gone particularly well in the CACP program.

    ____
    ____
    ____

30. Please describe anything that you think has gone poorly in the CACP program to help us make improvements in the future.

    ____
    ____
    ____

Thank you so much for your time and effort!
