# Supplementary material for: Exploring implementation and sustainability of a community paramedicine model to reduce hospitalizations: a pragmatic randomized trial
Source: BMC Health Serv Res. 2026 Apr 17;26:763. doi: 10.1186/s12913-026-14532-z (PMC13217778; doi:10.1186/s12913-026-14532-z)
Supplement: Supplementary file 3 — Supplementary Material 3 [file 12913_2026_14532_MOESM3_ESM.pdf]

INSTRUCTIONS: Please check the appropriate box or fill in the blank as indicated.

1. What is your role?
   - ☐ Physician
   - ☐ Nurse Practitioner (NP) or Physician Assisitant (PA)
   - ☐ Resident or fellow
   - ☐ Nurse
   - ☐ Case manager or social worker
   - ☐ Other: __________________ (please specify)
2. What is your primary practice location?
   - ☐ Rochester, Minnesota
   - ☐ Barron, Wisconsin
   - ☐ Bloomer, Wisconsin
   - ☐ Eau Claire, Wisconsin
   - ☐ Other:_____________________
3. Do you practice in the acute care setting (emergency department, hospital), outpatient setting, or both?
   - ☐ Acute setting only
   - ☐ Outpatient setting only
   - ☐ Both acute and outpatient settings
4. What are your <u>primary</u> clinical unit or department affiliations?
   - ☐ Cardiology
   - ☐ Emergency Department or Tele-Emergency Department
   - ☐ Inpatient medical services, including Hospital Internal Medicine services, Resident Medicine services, and inpatient Family Medicine services
   - ☐ Community Internal Medicine (including internal medicine residency clinic)
   - ☐ Senior Services programs (e.g., Care Transitions Program, Palliative Homebound Program)
   - ☐ Family Medicine (including family medicine residency clinic)
   - ☐ Vascular Medicine, including wound care
   - ☐ Gastroenterology
   - ☐ Nephrology
   - ☐ Neurology
   - ☐ Pulmonary Medicine
   - ☐ Hematology, medical oncology, or radiation oncology
   - ☐ Surgical service (*please specify*): ___________________________
   - ☐ Other (please specify): ______________________________
5. As part of your usual clinical responsibilities, do you perform home visits?
   - ☐ Yes
   - ☐ No

The following questions refer to the Care Anywhere with Community Paramedics (CACP) program. Patients were those randomized to home care with community paramedic support, after they were referred from the outpatient setting, emergency department, or hospital. It is part of a research study that began in February 2022. It does not include other community paramedic care services outside of the CACP program.

6. Have you referred any patients to the CACP program?
   - ☐ Yes

i. Approximately how many patients have you referred?
- ☐ 1-5
- ☐ 6-10
- ☐ 11-15
- ☐ 16-20
- ☐ 21 or more
- ☐ Not sure or don't know

☐ No

i. [If no] What is the primary reason(s)?
- ☐ Did not know about the program
- ☐ Did not understand the referral criteria or process
- ☐ Did not have any patients that met eligibilty criteria
- ☐ Do not think the program is appropriate for patients I see
- ☐ Other _____________________ (please specify)

7. Have you managed or clinically supported any patients enrolled in the *CACP* program, including those who were referred by others?

☐ Yes

i. Approximately how many patients have you managed?
- ☐ 1-5
- ☐ 6-10
- ☐ 11-15
- ☐ 16-20
- ☐ 21 or more
- ☐ Not sure or don't know

☐ No

[If the answers to Questions 6 and 7 are "No," end survey with message "Thank you for your time. We are interested in talking with people about their impressions of the Care Anywhere with Community Paramedics program. If you are willing to speak with a member of our study team, please click here and enter your email address. This information will only be used to contact you for this purpose; it will not be stored with your survey responses."]

How satisfied were you with each of the following aspects of the *CACP* program?
Instructions: For each item, please mark an "X" in the box that best describes how you feel or what is true for you.

		Very satisfied	Somewhat satisfied	Somewhat disatisfied	Very disatisfied	Not applicable
8.	Referral process	1☐	2☐	3☐	4☐	5☐
9.	Communication with the community paramedic leadership team	1☐	2☐	3☐	4☐	5☐
10	Communication with the community paramedics	1☐	2☐	3☐	4☐	5☐
11	Community paramedics' knowledge and	1☐	2☐	3☐	4☐	5☐

	understanding of patient care plans					
12	Community paramedics' implementation of patient care plans	1☐	2☐	3☐	4☐	5☐
13	Range of services offered by the *CACP* program	1☐	2☐	3☐	4☐	5☐
14	Geographic areas served by the *CACP* program	1☐	2☐	3☐	4☐	5☐
15	Patient safety of the *CACP* program	1☐	2☐	3☐	4☐	5☐
16	Efficiency of the *CACP* program	1☐	2☐	3☐	4☐	5☐
17	Effectiveness of CACP program in meeting patient goals	1☐	2☐	3☐	4☐	5☐
18	The *CACP* program overall	1☐	2☐	3☐	4☐	5☐

19. Could the *CACP* program be improved by expanding types of services community paramedics can provide in the home?
    - ☐ No, current CACP program services are adequate
    - ☐ Yes, the program would be improved if services were expanded
      Please specify what services could be added
      ____
      ____

20. How much do you think <u>patients</u> benefit from having the *CACP* program available to them?
    - ☐ A lot
    - ☐ Somewhat
    - ☐ A little bit
    - ☐ Not at all
    - ☐ Don't know

21. How much do you think <u>Mayo Clinic</u> benefits from having the *CACP* program available?
    - ☐ A lot
    - ☐ Somewhat
    - ☐ A little bit
    - ☐ Not at all
    - ☐ Don't know

22. How likely or unlikely are you to recommend the *CACP* program to providers?
    - ☐ Very likely
    - ☐ Somewhat likely
    - ☐ Not very likely
    - ☐ Not at all likely
    - ☐ Don't know
23. How likely or unlikely are you to refer patients to the *CACP* program in the future?
    - ☐ Very likely
    - ☐ Somewhat likely

- ☐ Not very likely
- ☐ Not at all likely
- ☐ Don't know

In the following questions, please rate the *Care Anywhere with Community Paramedics* program across a range of specific factors that affect program sustainability. Please respond to as many items as possible. If you truly feel you are not able to answer an item, you may select "NA." For each statement, select the number that best indicates the extent to which your practice has or does the following things.

24. **Organizational readiness**: Having the internal support and resources needed to effectively manage the program.

	To little or no extent						To a very great extent	Not able to answer
Organizational systems are in place to support the various needs of the *CACP* program	1	2	3	4	5	6	7	NA
The *CACP* program fits in well with the culture of the team	1	2	3	4	5	6	7	NA
The *CACP* program has feasible and sufficient resources (e.g., time, space, funding) to achieve its goals	1	2	3	4	5	6	7	NA
The *CACP* program has adequate staff to achieve its goals	1	2	3	4	5	6	7	NA
The *CACP* program is well integrated into the operations of the organization	1	2	3	4	5	6	7	NA

25. **Workflow integration**: Designing the program to fit into existing practices and technologies.

	To little or no extent						To a very great extent	Not able to answer
The *CACP* program is built into the clinical workflow	1	2	3	4	5	6	7	NA
The *CACP* program is easy for clinicians to use	1	2	3	4	5	6	7	NA
The *CACP* program integrates well with established clinical practices	1	2	3	4	5	6	7	NA
The *CACP* program aligns well with other clinical systems (e.g. electronic health record)	1	2	3	4	5	6	7	NA
The *CACP* program is designed to be used consistently	1	2	3	4	5	6	7	NA

26. **Outcomes and Effectiveness**: Understanding and measuring program outcomes and impact

	To little or no extent						To a very great extent	Not able to answer
The *CACP* program has evidence of beneficial outcomes	1	2	3	4	5	6	7	NA
The *CACP* program is associated with improvement in patient outcomes that are clinically meaningful	1	2	3	4	5	6	7	NA
The *CACP* program is clearly linked to positive health or clinical outcomes	1	2	3	4	5	6	7	NA
The *CACP* program is cost-effective	1	2	3	4	5	6	7	NA
The *CACP* program has clear advantages over alternatives	1	2	3	4	5	6	7	NA

27. Please describe anything that you think has gone particularly well in the *CACP* program.

____

____

____

28. Please describe anything that you think has gone poorly in the *CACP* program to help us make improvements in the future.

____

____

____

Thank you so much for your time and effort!
