# Supplementary material for: Exploring implementation and sustainability of a community paramedicine model to reduce hospitalizations: a pragmatic randomized trial
Source: BMC Health Serv Res. 2026 Apr 17;26:763. doi: 10.1186/s12913-026-14532-z (PMC13217778; doi:10.1186/s12913-026-14532-z)
Supplement: Supplementary file 4 — Supplementary Material 4 [file 12913_2026_14532_MOESM4_ESM.pdf]

INSTRUCTIONS: Please check the appropriate box or fill in the blank as indicated.

1. What is your primary role? (Select one)
   - ☐ Administrator
   - ☐ Social worker or case manager
   - ☐ Other: ___________________ (please specify)
2. What area are you primarily affiliated with? (Select one)
   - ☐ Emergency Department
   - ☐ Hospital
   - ☐ Ambulance Service
   - ☐ Outpatient practice
   - ☐ Other: ___________________ (please specify)
3. What is your primary location? (Select one)
   - ☐ Rochester, Minnesota
   - ☐ Barron, Wisconsin
   - ☐ Bloomer, Wisconsin
   - ☐ Other:______________________

The following questions refer to the Care Anywhere with Community Paramedics (CACP) program. Patients were those randomized to home care with community paramedic support, after they were referred from the outpatient setting, emergency department, or hospital. It is part of a research study that began in February 2022. It does not include other community paramedic care services outside of the CACP program.

How satisfied were you with each of the following aspects of the *CACP* program?
Instructions: For each item, please mark an "X" in the box that best describes how you feel or what is true for you.

		Very satisfied	Somewhat satisfied	Somewhat disatisfied	Very disatisfied	Not applicable
4.	Range of services offered by the *CACP* program	1☐	2☐	3☐	4☐	5☐
5.	Geographic areas served by the *CACP* program	1☐	2☐	3☐	4☐	5☐
6.	Patient safety of the *CACP* program	1☐	2☐	3☐	4☐	5☐
7.	Efficiency of the *CACP* program	1☐	2☐	3☐	4☐	5☐
8.	Effectiveness of the *CACP* program in meeting patient goals	1☐	2☐	3☐	4☐	5☐
9.	The *CACP* program overall	1☐	2☐	3☐	4☐	5☐

10. Could the CACP program be improved by expanding the types of services community paramedics can provide in the home?
    - ☐ No, current CACP program services are adequate
    - ☐ Yes, the program would be improved if services were expanded
      Please specify what services could be added:
      ____

11. How much do you think <u>patients</u> benefit from having the *CACP* program available to them?
- ☐ A lot
- ☐ Somewhat
- ☐ A little bit
- ☐ Not at all
- ☐ Don't know

12. How much do you think <u>Mayo Clinic</u> benefits from having the *CACP* program available?
- ☐ A lot
- ☐ Somewhat
- ☐ A little bit
- ☐ Not at all
- ☐ Don't know

13. How likely or unlikely are you to recommend the *CACP* program to providers?
- ☐ Very likely
- ☐ Somewhat likely
- ☐ Not very likely
- ☐ Not at all likely
- ☐ Don't know

In the following questions, please rate the *CACP* program across a range of specific factors that affect program sustainability. Please respond to as many items as possible. If you truly feel you are not able to answer an item, you may select "NA." For each statement, select the number that best indicates the extent to which your practice has or does the following things.

14. **Engaged staff and leadership**: Having supportive frontline staff and management within the program.

	To little or no extent						To a very great extent	Not able to answer
The *CACP* program engages leadership and staff throughout the process	1	2	3	4	5	6	7	NA
Clinical champions of the *CACP*program are recognized and respected	1	2	3	4	5	6	7	NA
The *CACP* program has engaged, ongoing champions	1	2	3	4	5	6	7	NA
The *CACP* program has a leadership team made of multiprofessional partnerships	1	2	3	4	5	6	7	NA
The *CACP* program has team-based collaboration and infrastructure	1	2	3	4	5	6	7	NA

15. **Engaged stakeholders**: Having external support and engagement for the program

	To little or no extent						To a very great extent	Not able to answer
The *CACP* program engages the patient and family members as stakeholders	1	2	3	4	5	6	7	NA
There is respect for all stakeholders involved in the *CACP* program	1	2	3	4	5	6	7	NA

The *CACP* program is valued by a diverse set of stakeholders	1	2	3	4	5	6	7	NA
The *CACP* program engages other medical teams and community partnerships as appropriate	1	2	3	4	5	6	7	NA
The *CACP* program team has the ability to respond to stakeholder feedback about the program	1	2	3	4	5	6	7	NA

16. **Organizational readiness**: Having the internal support and resources needed to effectively manage the program.

	To little or no extent						To a very great extent	Not able to answer
Organizational systems are in place to support the various needs of the *CACP* program	1	2	3	4	5	6	7	NA
The *CACP* program fits in well with the culture of the team	1	2	3	4	5	6	7	NA
The *CACP* program has feasible and sufficient resources (e.g., time, space, funding) to achieve its goals	1	2	3	4	5	6	7	NA
The *CACP* program has adequate staff to achieve its goals	1	2	3	4	5	6	7	NA
The *CACP* program is well integrated into the operations of the organization	1	2	3	4	5	6	7	NA

17. **Workflow integration**: Designing the program to fit into existing practices and technologies.

	To little or no extent						To a very great extent	Not able to answer
The *CACP* program is built into the clinical workflow	1	2	3	4	5	6	7	NA
The *CACP* program is easy for clinicians to use	1	2	3	4	5	6	7	NA
The *CACP* program integrates well with established clinical practices	1	2	3	4	5	6	7	NA
The *CACP* program aligns well with other clinical systems (e.g. electronic health record)	1	2	3	4	5	6	7	NA
The *CACP* program is designed to be used consistently	1	2	3	4	5	6	7	NA

18. **Implementation and training**: Using processes that guide the direction, goals, and strategies of the program.

	To little or no extent						To a very great extent	Not able to answer
The *CACP* program clearly outlines roles and responsibilities for all staff	1	2	3	4	5	6	7	NA
The reason for the *CACP* program is clearly communicated to and understood by all staff	1	2	3	4	5	6	7	NA
Staff receive ongoing coaching, feedback, and training	1	2	3	4	5	6	7	NA
CACP program implementation is guided by feedback from stakeholders	1	2	3	4	5	6	7	NA
The *CACP* program has ongoing education across professions	1	2	3	4	5	6	7	NA

19. **Monitoring and evaluation**: Assessing the program to inform planning and document results.

	To little or no extent						To a very great extent	Not able to answer
The *CACP* program has measurable process components, outcomes, and metrics	1	2	3	4	5	6	7	NA
Evaluation and monitoring of the *CACP* program are reviewed on a consistent basis	1	2	3	4	5	6	7	NA
The *CACP* program has clear documentation to guide process and outcome evaluation	1	2	3	4	5	6	7	NA
CACP program monitoring, evaluation, and outcomes data are routinely reported to the clinical care team	1	2	3	4	5	6	7	NA
The *CACP* program process components, outcomes, and metrics are easily assessed and audited	1	2	3	4	5	6	7	NA

20. **Outcomes and effectiveness**: Understanding and measuring program outcomes and impact.

	To little or no extent						To a very great extent	Not able to answer
The *CACP* program has evidence of beneficial outcomes	1	2	3	4	5	6	7	NA
The *CACP* program is associated with improvement in patient outcomes that are clinically meaningful	1	2	3	4	5	6	7	NA
The *CACP* program is clearly linked to positive health or clinical outcomes	1	2	3	4	5	6	7	NA
The *CACP* program is cost-effective	1	2	3	4	5	6	7	NA
The *CACP* program has clear advantages over alternatives	1	2	3	4	5	6	7	NA

21. Please describe anything that you think has gone particularly well in the *CACP*program.

____
____
____

22. Please describe anything that you think has gone poorly in the CACP program to help us make improvements in the future.

____
____
____

Thank you so much for your time and effort!
