# Supplementary material for: Exploring implementation and sustainability of a community paramedicine model to reduce hospitalizations: a pragmatic randomized trial
Source: BMC Health Serv Res. 2026 Apr 17;26:763. doi: 10.1186/s12913-026-14532-z (PMC13217778; doi:10.1186/s12913-026-14532-z)
Supplement: Supplementary file 5 — Supplementary Material 5 [file 12913_2026_14532_MOESM5_ESM.pdf]

WELCOME

Thank you for talking with me today. The purpose of this interview is to understand your experience with the Care Anywhere with Community Paramedics (CACP) program. *[If needed, clarify that the focus is on the CACP program and not the procedures of the clinical trial to evaluate it; Review oral consent.]*

INTRODUCTION

1. Before we talk about the program, can you tell me a bit about how you came to be in the program? *[Probe for the condition that caused their ER or hospital visit and the type of care they needed.]*

PROGRAM IMPRESSIONS + ENROLLMENT

2. How was the CACP program described to you?
    a. How well did you understand the options available, for example being in the emergency department or hospital or getting care at home from a community paramedic?
3. What types of things were you thinking about when deciding whether to get care in the emergency department or hospital versus at home with the community paramedics?
    a. What was most important to you when making that decision?
    b. What concerns did you have about receiving community paramedic care at home (rather than emergency department or hospital care), if any?
4. Who else was involved in that decision, and how did they help you think about the options?
5. How could we improve the process for how patients get enrolled in this program?
    a. How could we improve the information patients get about the program?

PROGRAM EFFECTIVENESS

6. Thinking back to when you enrolled in the program, what were some of the things that you were hoping the community paramedics could help you with?
7. Could you describe for me the types of care you got from the community paramedics?
    a. Could you walk me through an example or two of what happened during their visits?
8. How well do you think the program worked for you?
    a. What are some of the ways you benefited, if any?
    b. Were there times that you think having a community paramedic visit (or knowing one was coming up) helped you avoid a visit to the hospital, emergency department, or other healthcare facility? If so, could you tell me about that?
    c. Were there times that the community paramedic suggested you follow up with a health care provider, when you hadn't planned to do so? If so, could you tell me about that?
9. What aspects of the program could be improved so they work better for patients like you?
    a. Were there any times when you second-guessed your decision to get community paramedic care at home rather than stay in the hospital? If so, can you tell me about that? What could have been better in that situation?
10. How would you describe communication between you, the community paramedics, and other people involved in your care (e.g., family, friends, health care providers)?
    a. Could you give me an example of a time when communication worked really well?
    b. How about an example of when things did not go so smoothly?

PROGRAM SUSTAINABILTY

11. Should this program continue to be offered to patients like you? Why or why not?
    a. How important do you think it is that patients have access to a program like this?
12. Imagine that another community or hospital wanted to start a program like this. Would you suggest they do anything differently than this program has?

CLOSING

13. What else do you think we should know about the CACP program or how we can deliver the best care to patients in situations like yours?
