# Supplementary material for: Exploring implementation and sustainability of a community paramedicine model to reduce hospitalizations: a pragmatic randomized trial
Source: BMC Health Serv Res. 2026 Apr 17;26:763. doi: 10.1186/s12913-026-14532-z (PMC13217778; doi:10.1186/s12913-026-14532-z)
Supplement: Supplementary file 6 — Supplementary Material 6 [file 12913_2026_14532_MOESM6_ESM.pdf]

WELCOME

Thank you for talking with me today. The purpose of this interview is to understand your experience with the Care Anywhere with Community Paramedics (CACP) program. *[If needed, clarify that the focus is on the CACP program, not the pragmatic trial procedures evaluating it; Review oral consent.]*

INTRODUCTION

1. Before we talk about the program specifically, can you tell me a bit about your experience as a CP?
    a. What region (Rochester or NWWI) do you work in?
2. How does the CACP program align with the skills and focus of community paramedics?
    a. Do you feel that CPs are the right people for the work you are doing as part of CACP?
    b. How does the CACP program fit into your work alongside the other programs you are involved with? (Probe for amount of time/effort on this project vs other work)

PROGRAM NEED

3. How would you describe the reason for the CACP program?
    a. What gap does it fill?
    b. How would you describe its mission?

PROGRAM REFERRALS

4. Could you walk me through the process for referrals to the program? How is that going?
    a. What could be improved to make the process work better for you? For care teams?
5. How do patients typically respond when you first start working with them?

PROGRAM EFFECTIVENESS

6. How well do you think the program is meeting the needs of patients?
    a. Is current length of care for these patients appropriate?
    b. Can you describe for me an example of when the program worked really well?
7. What aspects of the program could be improved?
8. How well do you think the program works for all patients?
    a. Are there patients that the program isn't reaching or serving as well as it could?
9. How would you describe communication with the care teams?

CP TRAINING AND SUPPORT

10. What can you tell me about the training you received to deliver CACP services?
    a. Did you feel like the training prepared you well for this work? What could have been better?
    b. Were you ever uncomfortable with any aspect of the care or services that you were asked to deliver? (make sure they elaborate, using prompts if necessary)
11. How would you describe the support you got for delivering the program?
    a. How confident were you delivering the services?
12. What are the challenges of delivering a program like this (from your perspective)?
13. What are the most rewarding things?
14. If another organization was starting a program like this, how should they prepare CPs? How should they prepare other members of the care team?

PROGRAM SUSTAINABILTY

15. I want to ask you now about the future of the program. What are your thoughts on whether it should it continue in its current state or should be changed/scaled up?
16. What would be needed to make the ideal program in the future?

CLOSING

17. What else do you think we should know about the CACP program?
