# Supplementary material for: Exploring implementation and sustainability of a community paramedicine model to reduce hospitalizations: a pragmatic randomized trial
Source: BMC Health Serv Res. 2026 Apr 17;26:763. doi: 10.1186/s12913-026-14532-z (PMC13217778; doi:10.1186/s12913-026-14532-z)
Supplement: Supplementary file 7 — Supplementary Material 7 [file 12913_2026_14532_MOESM7_ESM.pdf]

WELCOME

Thank you for talking with me today. The purpose of this interview is to understand your experience with the *Care Anywhere with Community Paramedics* (CACP) program. *[If needed, clarify that the focus is on the CACP program and not the pragmatic trial procedures evaluating it; Review oral consent.]*

[With permission, start the recording.]

INTRODUCTION

1. Before we talk about the program specifically, can you tell me a bit about your role and how you interact with the CACP program? [*Probe for whether they are a clinician, bedside nurse, social worker, case manager, administrator (and in what unit/Department), Ambulance Service leadership, etc.; are they in the clinic, home care, ED, or hospital setting; did they refer patients to CACP program themselves vs. Had their patients referred vs. Cared for patients within the CACP program who were referred by others]*

PROGRAM NEED

2. How would you describe the reason for the CACP program?
   a. What gap does it fill?
3. How important is a program like this to patients, your practice, and to Mayo Clinic, if at all?
4. How would you describe support or buy-in among your leadership and clinical leadership more broadly? How about members of the care team, including clinicians who care for these patients?

PROGRAM REFERRALS [CLINICIANS]

5. Have you referred patients to the CACP program?
   a. If NO: Why not? What can be done to increase the likelihood that you will refer?
6. Could you walk me through the process for referrals to the program? How is that going? [*If participant said "no" to #5, ask them if they know how to refer and what the process is]*
   a. What could be improved to make the process work better for care teams?
7. *If #5 was "yes*" - What kinds of patients are you referring into the program?
8. How do you describe the program to patients? How do patients typically respond?
9. What kind of follow-up do you have (if any) with patients once enrolled into the CACP program?

PROGRAM EFFECTIVENESS

10. How well do you think the program is meeting the needs of patients?
    a. Can you describe for me an example of when the program worked really well?
11. How has program impacted (if at all) your care area? How well do you think the program is meeting the needs of clinicians (or the health system more broadly)?
12. What aspects of the program could be improved?
13. Are there patient populations you wish this program served or served better?
14. What are the biggest challenges to implementing a program such as this?
    a. Are there challenges unique to your practice or the patients you see?
15. How well do you think the program works for patients?
    a. Are there patients that the program isn't reaching or serving as well as it could?
16. How would you describe communication between the CPs and the care teams?
17. What reservations or concerns, if any, have you had about the program? Have these changed over time?

PROGRAM SUSTAINABILTY

18. I want to ask you now about the future of the program. What are your thoughts on whether it should it continue in its current state or should it be changed/scaled up?
19. What does the ideal program look like in the future?

CLOSING

20. What else do you think we should know about the CACP program?
